# Clergymen in Hospitals as Patient Companions during the COVID-19 Pandemic

**Published:** 2020-11

**Authors:** Reza Aminnejad, Hamed Shafiee, Reza Heidarifar, Rosa Alikhani

**Affiliations:** 1 Department of Anesthesiology and Critical Care, Qom University of Medical Sciences, Qom, Iran,; 2 Department of Anesthesiology and Critical Care, Shahid Beheshti University of Medical Sciences, Tehran, Iran,; 3 Department of Psychiatry, Psychosis Research Center, University of Social Welfare and Rehabilitation Sciences, Tehran, Iran.

As of December 2019, a new pandemic disease, called coronavirus disease-2019 (COVID-19), is spreading rapidly around the world (1). On February 19, Iran reported the first case of COVID-19 infection (2). Similar to previous outbreaks of infectious diseases, anxiety, besides other symptoms, was a common finding in hospitalized patients in Qom, Iran, which was also reported in patient companions (3). The unexpected workload, alongside limited human resources, particularly the nursing staff, was a common problem in most hospital wards during the early weeks of the outbreak.

A few weeks following the COVID-19 crisis, a volunteer group of clergymen, trained for primary personal protective measures, replaced the patient companions in Nekooei-Hedayati-Forghani Hospital. After one week, the patients, nurses, and physicians were asked if they were satisfied with the presence of volunteer clergymen as patient companions. The results showed that 92.9% of patients were satisfied with the presence of clergymen, while only 2% were unhappy. Fear of disease transmission to the volunteered companions and others (particularly families) was the only reason for the patients’ dissatisfaction. On the other hand, the reasons for the patients’ satisfaction with the presence of clergymen were overcoming the feeling of loneliness, reduction of fear and anxiety, and promotion of spiritual well-being.

In this study, 50.2% of nurses were satisfied with the presence of clergymen, whereas 32.5% were dissatisfied. The main reasons for the nurses’ dissatisfaction were the clergymen's interference in medical and nursing practices and fear of virus spread in the city. On the other hand, nurses who were satisfied with the presence of clergymen focused on the clergymen's role as patient companions, assisting patients in their religious affairs and care. Moreover, 75% of physicians were satisfied with the presence of clergymen in the hospital, and only 5.2% were dissatisfied. The main reason why physicians disagreed with the presence of clergymen was their lack of professionalism. However, physicians who agreed with the presence of clergymen believed that it could reduce the fear and anxiety of patients and promote their spiritual health.

Anxiety or panic is inevitable during outbreaks, and it is important to avoid them while managing such pandemics (4, 5). If necessary training is provided for volunteer clergymen regarding personal protection measures, their presence as patient companions appears to be effective during crises, which require as much emotional support as medical care; this is especially true in societies where people's religious beliefs are strong. Also, by using trained people as patient companions, we can spread accurate information in the community rather than fear (6).

## References

[1] Zhou F Yu T Du R Fan G Liu Y Liu Z Clinical course and risk factors for mortality of adult inpatients with COVID-19 in Wuhan, China: a retrospective cohort study. Lancet 2020; 395 (10229): 1054– 1062. 32171076 10.1016/S0140-6736(20)30566-3 PMC7270627

[2] Moradi G Piroozi B Mohamadi-Bolbanabad A Safari H Shokri A Rahimi R. Can judgments according to case fatality rate be correct all the time during epidemics? Estimated cases based on CFR in different scenarios and some lessons from early case fatality rate of coronavirus disease 2019 in Iran. Med J Islam Repub Iran 2020; 34: 26. 32551315 10.34171/mjiri.34.26 PMC7293816

[3] Mohammed A Sheikh TL Poggensee G Nguku P Olayinka A Ohuabunwo C Mental health in emergency response: lessons from Ebola. Lancet Psychiatry 2015; 2 (11): 955– 7. 26544738 10.1016/S2215-0366(15)00451-4

[4] Avoiding panic in a pandemic. Lancet 2009; 373 (9681): 2084. 10.1016/S0140-6736(09)61130-2 19541020

[5] The Lancet COVID-19: fighting panic with information. Lancet 2020; 395 (10224): 537. 32087777 10.1016/S0140-6736(20)30379-2 PMC7138040

[6] Gilman SL. Moral panic and pandemics. Lancet 2010; 375 (9729): 1866– 7. 20521345 10.1016/S0140-6736(10)60862-8 PMC7135638

